# Efficacy of psychodynamic short-term psychotherapy for depressed breast cancer patients: study protocol for a randomized controlled trial

**DOI:** 10.1186/1471-2407-12-578

**Published:** 2012-12-05

**Authors:** Rüdiger Zwerenz, Manfred E Beutel, Barbara H Imruck, Jörg Wiltink, Antje Haselbacher, Christian Ruckes, Heinz Schmidberger, Gerald Hoffmann, Marcus Schmidt, Uwe Köhler, Dagmar Langanke, Rolf-Dieter Kortmann, Susanne Kuhnt, Gregor Weißflog, Yvette Barthel, Katja Leuteritz, Elmar Brähler

**Affiliations:** 1 Department for Psychosomatic Medicine and Psychotherapy, University Medical Center Mainz, Untere Zahlbacher Str. 8, 55131, Mainz, Germany; 2 Interdisciplinary Center for Clinical Trials, University Medical Center Mainz, Langenbeckstr., 2, 55131, Mainz, Germany; 3 Clinic for Radiotherapy, University Medical Center Mainz, Langenbeckstr., 2, 55131, Mainz, Germany; 4 St. Josefs-Hospital, Breast Center, Beethovenstr., 20, 65189, Wiesbaden, Germany; 5 Clinic for Gynecology, Breast Center, University Medical Center Mainz, Langenbeckstr., 2, 55131, Mainz, Germany; 6 St. Georg-Hospital, Breast Center, Delitzscher Str. 141, 04129, Leipzig, Germany; 7 St. Elisabeth Hospital, Breast Center, Biedermannstr., 84, 04277, Leipzig, Germany; 8 Clinic for radiotherapy, University Medical Center Leipzig, Stephanstr., 9, 04103, Leipzig, Germany; 9 Department for Medical Psychology and Medical Sociology, University Leipzig, Philipp-Rosenthal-Str. 55, 04103, Leipzig, Germany

**Keywords:** Breast cancer, Depression, Short-term psychodynamic psychotherapy, Personality, Helping alliance, Quality of life

## Abstract

**Background:**

There is a lack of psychotherapeutic trials of treatments of comorbid depression in cancer patients. Our study determines the efficacy of a manualized short-term psychodynamic psychotherapy and predictors of outcome by personality and quality of the therapeutic relationship.

**Methods/design:**

Eligible breast cancer patients with comorbid depression are assigned to short-term psychodynamic psychotherapy (up to 20 + 5 sessions) or to treatment as usual (augmented by recommendation for counseling center and physician information). We plan to recruit a total of 180 patients (90 per arm) in two centers. Assessments are conducted pretreatment, after 6 (treatment termination) and 12 months (follow-up). The primary outcome measures are reduction of the depression score in the Hospital Anxiety and Depression Scale and remission of depression as assessed by means of the Structured Clinical Interview for DSM IV Disorders by independent, blinded assessors at treatment termination. Secondary outcomes refer to quality of life.

**Discussion:**

We investigate the efficacy of short-term psychodynamic psychotherapy in acute care and we aim to identify predictors for acceptance and success of treatment.

**Trial registration:**

ISRCTN96793588

## Background

Breast cancer is associated with multiple losses (e.g. regarding body image, sexuality, social relationships), strains (e.g. pain, fatigue) and threat to life. Depressive disorders are the most frequent mental comorbidities. The combined prevalence of major, minor depression and dysthymia in cancer patients was estimated at 22% [1,2]. In clinical routine, however, depression often escapes medical attention [3,4]. Without adequate treatment, depressive disorders in medically ill lead to substantial decrements of quality of life [5], longer inpatient treatment, prolonged work disability and inadequate illness behavior (e.g. lack of compliance) and even higher mortality [6].

Recently, there has been positive - somewhat limited - evidence for the effectiveness of pharmacological and psychotherapeutic treatments with randomized controlled trials (RCT’s) for depressed cancer patients, e.g. of cognitive behavior and problem-solving therapy for recently diagnosed, mildly to moderately depressed patients and of supportive-expressive group therapy for patients with advanced disease. Studies often suffer from methodological problems, such as selected or small samples, unclear or missing randomization, no manualization or control on treatment adherence. Unfortunately, only a minority of the trials has adequately assessed depression. A recent study ascertained clear preferences of cancer patients regarding speaking about their concerns and fears rather than accepting psychopharmacological treatment [7].

There has been increasing evidence supporting individual supportive-expressive psychotherapy as an effective short-term psychodynamic psychotherapy (STPP) for various mental disorders such as depression [8], generalized anxiety [9] and social phobia [10]. Based on the experience of developing a German manual [11] and training therapists in a multicenter RCT by two authors (MEB, AH), a specific treatment manual was developed for treating depression in breast cancer patients [12]. We assumed that psychodynamic treatment is suitable to deal with the intrapsychic and interpersonal conflicts generated by the experience of cancer. We also assumed that maladaptive interpersonal relationship patterns play a pivotal role in this context and that therapeutic changes of these patterns lead to remission or improvement of depression. Also, the combination of interpretative and supportive treatments renders supportive-expressive psychotherapy flexible to deal with crises in the course of a potentially life-threatening disease.

There is still a lack of knowledge on predictors of outcome of psychotherapy. Blatt & Zuroff [13] found that the success of short-term outpatient treatment depended mostly on two factors: the quality of the therapeutic relationship and patients’ pretreatment personality. In particular, those patients who were perfectionistic or self-critical before treatment improved less than those with low perfectionism. They obviously found it difficult to relate to their therapists in the time-limited treatment of depression. Thus, an additional issue of our trial is to determine the effects of personality and quality of the therapeutic relationship on treatment outcome.

## Methods/design

In a multicenter trial, patients are recruited in the centers of Mainz and Leipzig in close cooperation with gynecological and oncological centers in the respective regions (list of cooperating clinics cf. appendix). Assessments are done by independent, trained and supervised research-assistants, who are blind to the intervention. Quality assurance is performed by the independent Interdisciplinary Center for Clinical Trials with regular monitoring visits including source data verification of all randomized patients, control of patient existence and written consent of all screened patients. Monitoring is defined in a monitoring manual and all results of the monitoring are written down in monitor reports.

### Participants

Members of the oncological teams report eligible consecutive patients to trained research assistants. Following detailed information those who provide written consent with study participation are entered into the study and fill out the screening questionnaire. Patients are required to fulfill the test criteria on the Hospital Anxiety and Depression Scale (HADS, depression score ≥8) and a diagnosis of a depressive disorder by the Structured Clinical Interview for DSM IV Disorders (SCID-I). Patients are randomized to the intervention or control group only if a diagnosis of a depressive disorder is made based on the aforementioned criteria. Inclusion and exclusion criteria are listed in Table 1.

**Table 1:** Inclusion and exclusion criteria

*Inclusion criteria:*	· Diagnosis of breast cancer
	· Curative treatment
	· German language
	· Age 18-70
	· Depression score (HADS ≥ 8)
	· Depressive disorder according to SCID-I (ICD-10 diagnoses: depressive episode F32.-, recurrent depressive episode F33.-; Dysthymia F34.1, adjustment disorder F43.21)
	· Written consent with study participation
*Exclusion criteria:*	· Severe medical conditions (metastases, cognitive impairments) · Severe psychiatric disorders (psychotic disorder, risk of self-harm /suicide, acute substance related disorder, personality disorders except for cluster C, organic mental disorder) · Concurrent psychotherapeutic treatment

### Intervention

The intervention group is offered a manualized STPP adapting the approach of Luborsky et al. [8,14] to the specific needs of depressed breast cancer patients [12]. Following the concept of the Core Conflict Relationship Theme (CCRT), depression is conceptualized in the context of intrapsychic and interpersonal conflicts. The CCRT is the treatment focus characterizing a maladaptive relationship pattern consisting of a wish, the response of the other and of the self. Five pre-treatment sessions include history taking (also the basis for formal application to the health insurance) and eliciting relationship episodes in the relationship interview to formulate the CCRT. The treatment agreement is established in one of the sessions. The therapist shares the CCRT with the patient, informs him about depression and about the treatment rationale in order to engage him in treatment. In the *initial* treatment phase (sessions 1–6), the therapist encourages building a positive alliance and links depressive symptoms to the CCRT. In the *middle* phase (sessions 7 to 12), the therapist refines the CCRT based on further relationship episodes from past, ongoing relationships and the relationship to the therapist. The vulnerability to depression is reduced when its dynamic can be understood from different points of view. In the *termination phase* (13 to 18) the therapist focuses on the issues of separation and the patient’s ability to transfer the tools acquired in treatment to daily life. *Booster sessions* (19, 20) help to consolidate treatment progress. Treatment principles explicitly specified consider both the depressed state (e.g. dealing with helplessness/ hopelessness, anger, suicidality, negative attribution style) and the chronic and life-threatening disease (e.g. realistic treatment goals, here and now perspective, resource orientation). Psychodynamic therapists are certified or advanced trainees with psycho-oncological experience. They are trained in the manualized treatment during workshops before starting treatments and are regularly supervised during the treatments. For the supervision, each patient receiving the STPP intervention is presented at three time points (formulation of the CCRT after the relationship interview, in the middle phase of STPP and in the termination phase of STPP). Supervision is free of charge for the participating psychotherapists. Each session is videotaped. For the evaluation of treatment adherence and competence cf. assessment.

### Assessments

At baseline, patients fill out the HADS [15] in the German version (HADS-D) [16], a standardized questionnaire devised for the assessment of depression and anxiety in somatic illness with 14 items. Quality of life is assessed by the generic 30-item questionnaire of the European Organization for Research and Treatment of Cancer Quality of Life Questionnaire - Core 30 (EORTC QLQ-C30) [17] differentiating a global health status, functioning (physical, role, emotional, cognitive, social) and symptoms (e.g. fatigue, nausea and vomiting, pain etc.). Furthermore, the breast cancer specific quality of life module (EORTC QLQ-BR23) [18] which consists of functional scales (body image, sexual functioning, sexual enjoyment, future perspective) and symptom scales (system therapy side effects, breast symptoms, arm symptoms and upset by hair loss) is used. The validated German version of the Depressive Experience Questionnaire (DEQ) [19] is used to identify prognostically relevant personality dimensions of dependency (loneliness, helplessness, fear of rejection), perfectionism or self-criticism (worthlessness, failure, guilt, critical self-monitoring) and self-efficacy (ambitious, competitive and self-reliant stance). The Multidimensional Fatigue Inventory (MFI) [20] is a 20 item self-report instrument covering the dimensions general fatigue, physical fatigue, reduced activity, reduced motivation and mental fatigue. The German version [21] of the Helping Alliance Questionnaire (HAQ) [14] an 11-item rating scale for assessing perceptions of the therapeutic relationship and process is filled out by the patient and therapist at the end of psychotherapy. The Structured Clinical Interview for DSM-IV [22] is used for standardized (‘gold standard’) assessment of axis I diagnoses. The PACS-SE (Penn Adherence/Competence Scale) with 45 Items [23] is used in the German version [24] for independent assessment of therapist adherence and competence based on randomly selected videotaped therapy sessions.

### Objectives

The main purpose of the ongoing trial is to determine the efficacy of the manualized STPP regarding remission of depression in breast cancer patients. Secondary outcomes refer to changes of quality of life and to the effect of subtype of depression on treatment outcome.

Based on non-responder analyses we intend to answer the question, which patients do not accept psychotherapeutic treatment for what reason and which characteristics (e.g. age, marital, social status, illness variables, type of depression) have an impact on treatment outcome?

### Hypotheses

Primary outcome:

1) Higher rate of remission in STPP vs. ‘treatment as usual’ (TAU) at treatment termination.

Secondary outcomes:

2) Six months after the end of treatment lower rate of depression and a higher quality of life among the STPP group compared to TAU.

3) Better outcome in dependent/anaclitic vs. self-critical/ perfectionistic depression (DEQ).

### Study design

**Figure 1:**
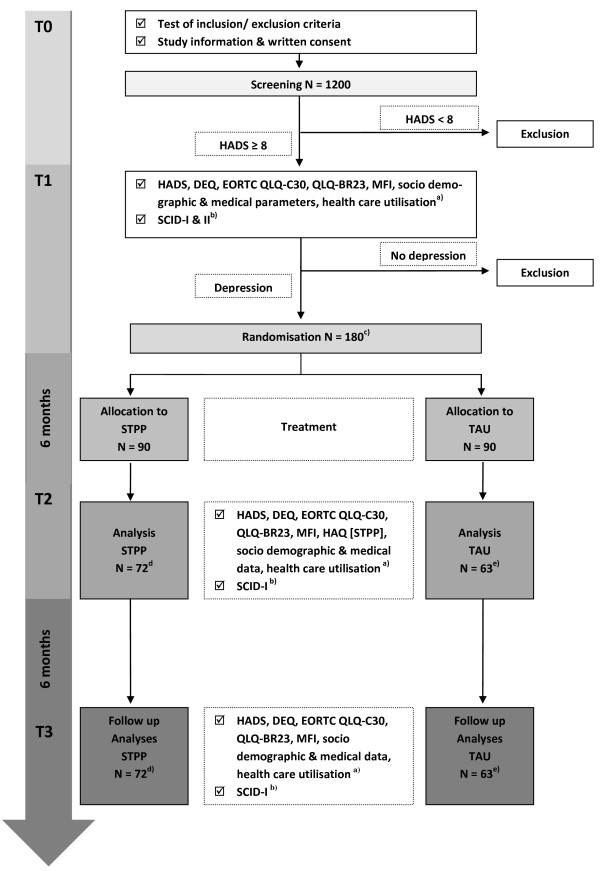
**Study design and patient recruitment (N = projected numbers).** Notes: a) Questionnaires filled out by the patients; b) Structured Clinical Interview for DSM-IV applied by trained interviewers; c) HADS score for depression ≥ 8 & SCID-I diagnosis estimated in 20% of the cases (N = 240 eligible patients) of whom 75% are expected to be consent with randomization; d) Expected completer 80%, e) Expected completer 70%.

In the multicenter randomized prospective trial breast cancer patients with comorbid depression who fulfill inclusion criteria either get STPP or TAU. Figure 1 gives an overview of the study design, time-points of assessments measures used and projected numbers.

We plan to recruit N = 90 participants per group. To ensure adequate randomization stratified for the center, the random assignment to STPP or TAU was performed separately by research staff from the responsible center (not engaged in the project) using a computer-generated [25] number series of random length (contained within closed, opaque envelopes.).

STPP entails five pre-treatment and up to 20 therapeutic sessions over a period of six months. In each center a close collaboration was established with about 10 psychodynamic psychotherapists in private practice who were trained according to the manual and supervised on a regular basis. Intensive training (at least 2 x 2 hours) is performed in a group format by the respective local study center prior to entering the first patient. Supervision is performed on a monthly basis (3 hours) in the group. In order to assure adherence to the manual, it is recommended that therapists present patients in supervision three times (initial formulation of CCRT based on relationship episode interview, middle and termination phase of treatment).

Patients randomized to the TAU condition are not offered STPP, but obtain written information on local cancer counseling centers and get written information on psychodiagnostic findings to their general practitioner who may then initiate antidepressant treatment (‘as usual’). Utilization of medical, psychotherapeutic and psychopharmacological treatment is documented throughout the study.

### Outcome

Remission of depression is defined by reduction of the HADS depression-score (at least by 2 points) and remission of depression (SCID-I-diagnosis) at treatment termination.

### Sample size calculation

While planning this study no meaningful published data on remission rates for STPP vs. TAU for the treatment of depression in breast cancer were available. For STPP of depression remission rates between 45% and 70% at post-therapy assessment were published [26], while remission rates in primary-care settings (comparable to TAU) ranged between 20% and about 50% (6 months) [27-29]. For patients with complex medical illness like cancer and co-morbid depressive disorders we expect lower remission rates for TAU and also for STPP. For this highly burdened population we pragmatically expected a spontaneous remission rate of 25% for TAU and 50% for STPP in our sample size calculation.

In order to identify group differences of 25% (25% remission in TAU vs. 50% in STPP) as based on χ2-Test (two-tailed) and a power of 0.80 a total sample of N = 156 is required, taking into account a slightly higher dropout rate of 30% in the TAU group vs. a 20% in STPP.

### Statistical analyses

The primary endpoint remission after the treatment phase will be analyzed by a logistic regression with fixed effects for treatment and center and the baseline HADS value as covariate. The primary population for analysis will be the population intended to treat (ITT) consisting of all randomized patients. Dropouts will be regarded as non-remitters. For sensitivity the analysis will be repeated for the completers of treatment population. Additionally, the single components (HADS improvement of ≥2 points, remission according to SCID) will be analyzed separately by the same analysis model as the combined endpoint.

Secondary analyses include the remissions at the end of the follow-up. Remissions at the end of the follow-up will be evaluated accordingly. The quality of life (QoL) questionnaires will be analyzed by an analysis of covariance (ANCOVA) model with fixed effects for treatment and center and the baseline values (HADS depression score & Qol) as covariates. It is expected that the QoL questionnaires will not be linear across the study. Therefore QoL questionnaires after the follow-up will be evaluated by a linear model with fixed effects for treatment, center and time. There will be an indicator variable for the post treatment visit. The HADS value at baseline will serve as a covariate. For QoL data the observed values will be analyzed. However, if the number of missing data is more than 10%, the data will be evaluated after multiple imputation of the data by a Markov chain Monte Carlo (MCMC) method. The impact of moderator variables (age, marital, social status, illness variables, type of depression) is tested in regression analyses.

### Safety aspects and medical complications

Safety parameters will comprise newly occurring mental diagnoses and all serious adverse events that are reported during and up to six months after treatment.

The recording of adverse events will be restricted to psychological conditions. Formally, they are defined as any disorder classified by the International Classification of Diseases F00 - F99. A serious adverse event (SAE) is an adverse event that may occur at any time of the treatment phase or up to 6 months after end of treatment: results in death; is life-threatening; requires subject hospitalization or prolongation of existing hospitalization; results in persistent or significant disability/incapacity; is a congenital anomaly/birth defect.

Any SAE (according to the study specific definition) reported by the subject or detected by the local investigator will be collected during the trial and must be documented in case report forms. ICD-10 will be used by the local investigator to code the event. The clinical course of the SAE will be followed until it has changed to a stable condition or until end of follow-up phase, whatever comes first. In case of SAEs the Ethics Committee will be informed within 24 hours after the SAE becomes known.

### Ethical issues

The final study protocol and the final version of the written informed consent form were approved by the Ethics Committee of the Federal State of Rhineland-Palatinate in Germany [reference number 837.380.06 (5478)] and the Ethics Committee of the University of Leipzig [reference number 218–2007]. The procedure set out in this protocol, pertaining to the conduct, evaluation, and documentation of this trial, were designed to ensure that all persons involved in the trial abide by Good Clinical Practice and the ethical principles described in the current revision of the Declaration of Helsinki [30]. The trial will be carried out in keeping with local legal and regulatory requirements. Before being admitted to the clinical trial, patients must consent to participate after the nature, scope, and possible consequences of the clinical trial have been explained in a form understandable to them. The patients must give written informed consent to participate in the study including their consent to publish.

## Discussion

This is the first trial to determine the efficacy of a manualized STPP regarding remission of depression in breast cancer patients. Secondary outcomes refer to changes of quality of life and to the effect of subtype of depression on treatment outcome. Unlike previous studies we required a diagnosis of depression for study entry and we used remission of depression as a clinically relevant outcome criterion.

As we wish to contribute to evidence-based psycho-oncological care we chose as a control condition treatment as usual. We are aware that quality of care for the individual patient in the control condition may vary. However, we actually perform an augmented TAU condition by referring patients to a collaborating cancer counseling center which may provide individual counseling or further referral. We also take great care to send written and detailed findings on the comorbid depression diagnosis to the general practitioner of all the patients (IG and CG) who have given their written consent to do so. We make sure that we carefully assess the actual health care utilization by all patients during both follow-ups.

As we planned to provide an intervention for acute care, we recruit patients who are still in active treatment (chemotherapy, radiotherapy). Recruiting in the major local clinics we make sure that we could assess and contact the breast cancer patients with comorbid depression. We are aware, however, that a substantial proportion of patients would refuse study participation feeling strongly burdened by ongoing treatment. Based on non-responder analyses we intend to answer the question, which patients do not accept psychotherapeutic treatment for what reason [31]. Including the important dimensions of therapeutic alliance and personality dimensions shaping the experience and expression of depression we also ascertain who benefits most from treatment.

Previous studies of STPP adapting the approach of Luborsky et al. [8,14] found no sex differences regarding treatment response [9]. Supportive-expressive psychotherapy has proven a robust model of short-term treatment for a diverse range of mental disorders. We therefore anticipate that it will be possible to transfer the treatment manual also to other kinds of cancer with comorbid depression less frequently studied - particularly in men.

## Abbreviations

CCRT: Core Conflict Relationship Theme; DEQ: Depressive Experience Questionnaire; DSM-IV: Diagnostic and Statistical Manual of Mental Disorders (4^th^ revision); EORTC QLQ-C30: European Organization for Research and Treatment of Cancer Quality of Life Questionnaire - Core 30; HADS: Hospital Anxiety and Depression Scale; HAQ: Helping Alliance Questionnaire (patient-, therapist-form); ICD-10: International Classification of Diseases (10^th^ revision); ITT: Intention to treat; MFI: Multidimensional Fatigue Inventory; PACS-SE: Penn Adherence/Competence Scale; EORTC QLQ-BR23: Breast cancer specific quality of life module; QoL: Quality of life; RCT: Randomized controlled trials; SAE: Serious adverse event; SCID I: II, Structured Clinical Interview according to DSM-IV; STPP: Short-Term Psychodynamic Psychotherapy; TAU: Treatment as usual.

## Competing interests

The authors declare that they have no competing interests.

## Authors’ contributions

The study design and assessments were conceptualized and developed by MEB, RS, AH, RZ, BHI, YB, SK, EB. Implementation and conduction of the study was coordinated by BHI, RZ, YB, SK and KL. RZ and MEB wrote an outline of the paper, which was carefully revised, edited and discussed by JW, CR, KL, YB, GW and EB. All authors read and approved the final manuscript.

## Pre-publication history

The pre-publication history for this paper can be accessed here:

http://www.biomedcentral.com/1471-2407/12/578/prepub
